# Endogenous Lipid Carriers—Bench-to-Bedside Roadblocks in Production and Drug Loading of Exosomes

**DOI:** 10.3390/ph16030421

**Published:** 2023-03-10

**Authors:** Terjahna Richards, Himaxi Patel, Ketan Patel, Frank Schanne

**Affiliations:** College of Pharmacy & Health Sciences, St. John’s University, Queens, NY 11439, USA

**Keywords:** exosome, drug loading, exosomal delivery, large-scale production

## Abstract

Exosomes are cell-derived, nano-sized extracellular vesicles comprising a lipid bilayer membrane that encapsulates several biological components, such as nucleic acids, lipids, and proteins. The role of exosomes in cell–cell communication and cargo transport has made them promising candidates in drug delivery for an array of diseases. Despite several research and review papers describing the salient features of exosomes as nanocarriers for drug delivery, there are no FDA-approved commercial therapeutics based on exosomes. Several fundamental challenges, such as the large-scale production and reproducibility of batches, have hindered the bench-to-bedside translation of exosomes. In fact, compatibility and poor drug loading sabotage the possibility of delivering several drug molecules. This review provides an overview of the challenges and summarizes the potential solutions/approaches to facilitate the clinical development of exosomal nanocarriers.

## 1. Introduction

**Figure 1:**
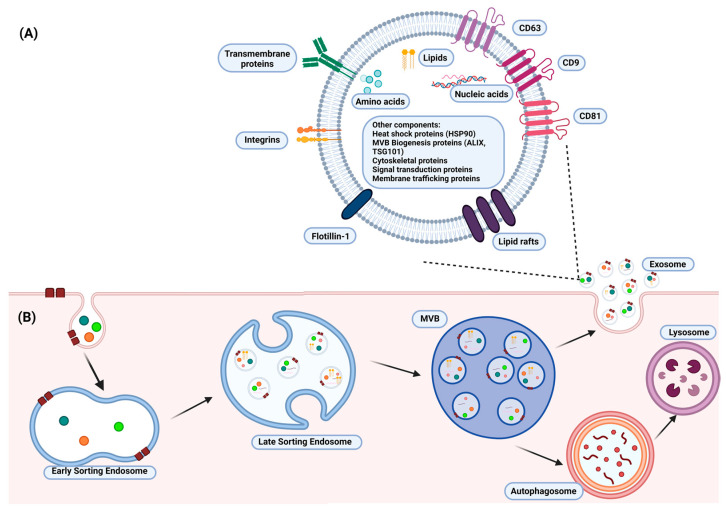
A schematic illustration of (**A**) the structure of a typical exosome and (**B**) the formation of exosomes. The structure of the exosome consists of intracellular (lipids, nucleic acids, and proteins) and extracellular components (tetraspanins, lipid rafts, flotillin-1, and transmembrane proteins), which assist in its characterization and many cellular functions. Exosomes are produced from a multivesicular body (MVB), which arises from a late-sorting endosome (LSE). The biogenesis of exosomes also involves specific sorting mechanisms responsible for transportation and an intraluminal vesicle (ILV) budding in the LSE. The illustration was created with BioRender.com (https://app.biorender.com; accessed on 16 January 2023).

Exosomes, a subclass of extracellular vesicles, are lipid bilayer vesicles with an average diameter of 100 nm that are secreted by all cell types. Exosomes consist of a multitude of extracellular and intracellular bioactive compounds, which play a crucial role in cellular communication and cargo transport [1,2]. Extracellular components include tetraspanins (CD9, CD81, CD63), lipid rafts, flotillin-1, integrins, and transmembrane proteins (Figure 1A). This is in contrast with intracellular components, which include lipids, nucleic acids, and various proteins, such as cytoskeleton proteins and heat shock proteins (Figure 1A). Exosomes are produced from a specific bilayer organelle called a multivesicular body (MVB) (Figure 1B). The formation of the MVB includes several phases: (1) inward budding of the cell membrane, (2) formation of the early-sorting endosome (ESE), (3) formation of the late-sorting endosome (LSE), where exosome precursors called intraluminal vesicles (ILVs) are germinating, and (4) transformation of the LSE to a mature MVB (Figure 1B) [1,2]. Exosome biogenesis is also associated with specific sorting mechanisms, such as the endosomal sorting complex responsible for transport (ESCRT), which assists in cargo sequestration and ILV budding. The diversity shown in exosome development and characteristics aids in their isolation from other extracellular vesicles.

Exosomes possess favorable pharmacokinetic properties, biocompatibility, and tissue-targeting abilities due to their phospholipid bilayer structure and various bioactive components, such as mRNAs, microRNAs, cytokines, chemokines, and immunomodulatory compounds. Moreover, exosomes have the ability to suppress inflammation, regulate cell proliferation, and deliver biotherapeutics [3,4,5,6]. Nevertheless, the feasibility of exosomes as therapeutic agents remains limited, which may be attributed to low exosome production and poor drug loading. However, in recent years, there has been an increase in research devoted to overcoming the limitations of exosome-based therapies. The incorporation of alternative exosome sources, upstream strategies, and downstream strategies have been used to improve the yield of exosomes. Additionally, adjustments have been made to several commonly used drug-loading techniques, and new procedures have been developed to improve the drug loading of exosomes. This review summarizes the challenges and provides potential solutions for exosome production and drug loading to facilitate the clinical development of exosome nanocarriers. 

## 2. Exosomal Drug Delivery: Challenges

### 2.1. Exosome Production and Isolation

Although exosomes have been shown to possess invaluable qualities for use in nanomedicine, their low production rate in unaltered cell cultures remains a key challenge, preventing bench-to-bedside use. In addition to the low production of exosomes, large variability in their size also exists, resulting in a lack of reproducibility in batches [1,7,8]. Consequently, the need remains to develop techniques that increase exosome production, maintain constant morphology, and limit any negative impact on cell cultures. It is worth mentioning that attention should be given to the shelf-life, stability, and storage of exosomes in their use as therapeutics.

Exosome isolation methods are important to increase the yield of exosomes. The currently available techniques for exosome isolation are based on their chemical, physical, and immunoaffinity assays and adapted from previous methods used for the isolation of viruses and other vesicles. Ultracentrifugation, the gold standard for exosome isolation, is one of the most applied techniques. However, its low recovery rate, low purity, and time-consuming process are not ideal for the implementation of exosomes in nanomedicine. Other commonly used techniques include polymer-based precipitation, ultrafiltration, size-exclusion chromatography, immunoaffinity chromatography, and microfluidics (Table 1). It is worth noting that the method of exosome isolation used may affect the yield and characteristics such as the size, structure, and biofunction of exosomes [3,9]. Thus, modifications to current methods and the development of new procedures are required to increase the yield and purity of exosomes. 

Preservation is important for maintaining the biological functions of exosomes and ensuring the ease of their transportation and clinical use [2,25]. Currently, there are various techniques used to improve the storage, shelf-life, and stability of exosomes. These include freeze-drying, spray-drying, and cryopreservation [2]. Freeze-drying, which is divided into three stages—pre-freezing, sublimation drying, and analytical drying, leads to the cooling of liquid components, followed by freezing. Exosomes that are stored using this method maintain their original activity but are exposed to membrane damage. Spray-drying involves the use of atomization pressure and hot air for the storage of exosomes, which may affect the stability of these extracellular vesicles. Cryopreservation, which is conducted at −80 °C, is the most commonly used method [2]. It enables the short-term storage of exosomes through the reduction of biochemical activity so that functional stability can be maintained. Furthermore, several studies have suggested that the addition of cryoprotectants, such as trehalose or DMSO, is mildly protective in maintaining exosome ability [2,26]. Despite these benefits, cryopreservation is associated with membrane destabilization and protein degradation, which may affect the therapeutic function of exosomes. In addition, the storage of exosomes for four days at −80 °C has been noted to affect their morphology, and at 28 days, their biological activity starts to be affected [2,27,28]. Therefore, further analysis of the storage, stability, and shelf-life of exosomes is of utmost importance.

### 2.2. Exosome Drug Loading

In addition to low production and reproducibility, another key challenge in the use of exosomes in nanomedicine is poor drug loading. Exosomes have shown favorable biocompatibility and therapeutic targeting abilities, thus making them valuable as a potential drug delivery tool. However, several factors, such as the exosome size, the pharmacokinetics of the drug, and the drug size, may hinder the efficiency of drug loading and require more specialized techniques [6,8,29,30]. For example, an exosome of a larger size may be loaded with a drug more easily than one with a smaller size. Moreover, a lipid-soluble drug may be loaded more quickly than a water-soluble drug. The exosomal structure, coupled with a therapeutic drug, requires careful consideration in the drug-loading process. Thus, new and improved procedures should be developed to enhance the effectiveness of exosome drug loading.

Drug-loading techniques can be categorized based on the time of implementation—pre-secretory or post-secretory [2]. Pre-secretory drug loading involves the loading of drugs before the development of the exosome, whereas post-secretory refers to drug loading after exosome development. Most drug-loading techniques are post-secretory and include sonication, electroporation, passive incubation, and the freeze–thaw cycle (Table 2). 

The advantages and disadvantages of each drug-loading technique (Table 2) depend on the experimental settings, type of drug, and source of exosomes. Passive incubation, for example, is a simple technique that involves the incubation of purified exosomes with drugs to allow for incorporation into the exosome membrane [36,38,45]. For example, the small molecule doxorubicin was passively loaded into exosomes by Wei et al. for osteosarcoma treatment [46]. Passive incubation is primarily used due to its excellent performance in the incorporation of hydrophobic compounds, such as curcumin [38]. Hydrophobic compounds can interact with the lipid bilayer of the exosome more effectively than hydrophilic compounds, and thus, can be incorporated into the exosome. The loading of hydrophilic compounds can be enhanced with the addition of the mild surfactant saponin, which, according to studies, induces transient membrane destabilization and can be used for the loading of large compounds (>200 kDa) [3,43]. However, the use of saponins may also affect biomolecules, and thus, requires purification before clinical use. Mechanical methods, such as sonication, nanoporation, and electroporation have been shown to successfully load small molecules and macromolecules into exosomes [8,30,47,48]. Research conducted by C Liu et al., for example, incorporated one of the mechanical techniques, i.e., microfluidic sonication, to effectively load PLGA into exosomes isolated from a human lung carcinoma cell line (A549) [33]. In addition, a study by Rodriguez-Morales et al. used electroporation to effectively produce insulin-loaded exosomes for the treatment of diabetes mellitus [35]. It is worth noting that these post-secretory drug-loading techniques may affect the proteins and nucleic acid drugs that are incorporated into the exosome and the structure of the exosome. The complexity of some of these methods, such as nanoporation, may render large-scale use in a clinical setting difficult. Consequently, there is a great need for effective drug-loading techniques that can be implemented on a large-scale in nanomedicine.

## 3. Exosomal Drug Delivery: Solutions

### 3.1. Exosome Production and Isolation

**Figure 2:**
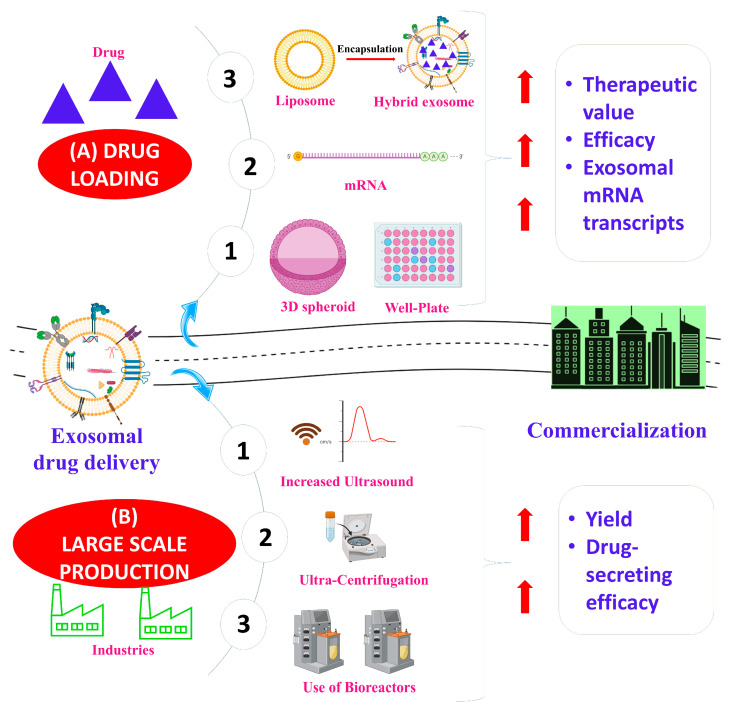
Key challenges in exosome production—(**A**) drug loading and (**B**) exosome production—are summarized. The therapeutic value and efficacy of the drug loading of exosomes can be improved through several methods, including the use of exosome–liposome hybrids and gene editing. The yield and drug-secreting efficacy of exosome production can be improved through ultrasound, ultracentrifugation, and the use of bioreactors. Improvements in drug loading and exosome production aid in the commercialization of exosome-based therapeutics. The illustration was created with BioRender.com (https://app.biorender.com; accessed on 16 January 2023).

For the use of exosomes in a clinical setting, large-scale production is required. Research has identified the important areas that should be considered in addressing this issue. These include the selection of exosome sources and modifications (upstream and/or downstream) (Figure 2).

#### 3.1.1. Source Selection

Exosomes can be produced from human and non-human sources. Human sources involve exosome production and isolation from the cells and fluids of the body. For example, stem cells have been shown to increase exosome production and provide larger-sized extracellular vesicles—a characteristic important for effective drug loading [49,50,51]. Research by Haraszti et. al. noted that human umbilical cord stem cells produce approximately four-fold larger-sized exosomes than bone marrow mesenchymal stem cells [52]. Other cell types need to be studied to evaluate exosome production and the therapeutic ability of these extracellular vesicles. This may prove to be beneficial in increasing exosome yield and improving reproducibility across batches. 

The non-human sources, which arose from the increasing demand for exosome-based therapeutics, include prokaryotes (Gram-positive bacteria and Gram-negative bacteria) [53,54,55], bovine milk [38,56], parasitic helminths [57], plants [58], and protists [59,60]. Compared to human sources, these types of exosome sources are versatile, and hence, more easily altered in upstream and downstream modifications than the human sources. Their versatility is beneficial to the large-scale production and use of exosomes in vaccines, therapeutics, and drug delivery. For example, the vesicles of the Gram-negative *Neisseria meningitidis* were approved for use in vaccines [61]. However, a critical setback is that these exosomes can be immunogenic or allergenic depending on the administration route, dosage, and dose frequency. Furthermore, several studies have noted that the variability in the upstream and downstream modifications used to generate these exosomes introduces experimental bias, which consequently, affects the immunological outcomes [54,55]. In general, it can be stated that the source from which exosomes are derived may affect their production and properties, which may cause variable therapeutic outcomes in production. As a result, careful consideration should be taken in selecting the appropriate source.

#### 3.1.2. Upstream Modifications

Exosome production can be influenced by modifications to the cell culture conditions. This may include appropriate cell selection and changes to the culture medium, the environmental parameters, and the method of cultivation. However, the alteration of cell culture conditions may affect the structure of exosomes and the productivity of the cultured cells.

##### Soluble Factors 

The addition of soluble factors to the cell culture medium can be used to increase exosome production (Table 3). Bioactive cytokines, such as lipopolysaccharide (LPS) [62], N-methyldopamine [63], norepinephrine [63], serotonin [64], adiponectin [65], adenosine triphosphate (ATP) [66], Wnt3a [67], calcium (Ca^2+^) ionophores [64], and plant ceramide [68] have been used in research to increase exosome production (Table 3). Furthermore, the upregulation of NadB, syndecan 4, and six-transmembrane epithelial antigen of prostate 3 (STEAP3) has increased the exosomes produced in cell cultures [7,69,70]. Research has shown that the genetic overexpressions of tetraspanin CD9 and hypoxia-induced factor 1α (HIFα) have increased exosome production by 2.4- and 2.2-fold, respectively [71,72,73]. However, the property and therapeutic efficacy of exosomes may be affected by the use of soluble factors. As a result, there is hesitancy in the use of soluble factors to preserve the cell culture environment. 

##### Chemical and Physical Stimulation

Alterations to the cell culture environment may cause cellular adaptation and consequently lead to changes in the characteristics of cells, thus resulting in increased exosome production. On this basis, chemical or physical damage-mimetic micro-environments have been created to increase exosome production and subsequent therapeutic functions (Table 3).

Chemical stimulations, such as hypoxia, have been shown to produce exosomes with enhanced therapeutic effects [71,72,74,75]. Serum deprivation, another example of chemical stimulation, exhibits variable effects on exosome production. Moreover, studies have revealed that the ability of serum deprivation to increase exosome production depends on the cellular origin [76,90]. Physical simulation involving flow and stretching factors, such as bioreactors, can increase exosome production. Studies involving the use of bioreactors have shown elevated exosome production by up to 37-fold [77,78]. Ambattu et al. employed another technique, where cells were stimulated with high-frequency ultrasound, resulting in an 8-10-fold increase in exosome production [79]. It is worth noting that the use of chemical and physical stimulations may affect the cellular characteristics.

##### 3D Culture

The mode of cultivation, such as 3D culture, can be used to expand the cell culture area, and exosome production can be increased by continually applying a shear force to the enlarged area (Table 3). Methods of 3D culture include the hanging drop in a 3D spheroid culture and the microcarrier-based suspension culture. The efficiency of the hanging-drop technique plateaued after a 2–3-fold increase [80]. The microcarrier-based suspension culture, an extensively used suitable method for 3D culture, showed increased exosome production of approximately 20-fold [81,82,83]. Additionally, in combination with a tangential flow filtration system, exosome production was further increased by 140-fold [52]. Recently, Patel et. al. cultured cells on a 3D-printed hollow fibrillar scaffold with a complementary perfusion system and reported a 100-fold increase in exosome production [84]. However, later experiments conducted by Patel et al. demonstrated that the structure and components of exosomes were substantially affected. It was noted that the extracellular components were significantly decreased, and the complex process of the 3D printing scaffold required special training. In another study, Burns et. al. developed a low-shear technique for 3D cell cultivation that was reported to maintain cell viability, purity, and phenotype [85]. Notably, in 3D cell cultivation, the conditions of the cell culture and the shear force applied requires careful evaluation to limit the effects on cell viability and phenotype. 

##### Biomaterials

Biomaterials could improve exosome productivity and their therapeutic ability by creating a special microenvironment for cellular interaction. Biomaterials used in cell culture include nitric oxide-releasing polymer [86], lithium-incorporated bioactive glass ceramic [87], iron oxide-coated polylactic-co-glycolic acid (PLGA) nanoparticles [88], and bioglass [89] (Table 3). Kojima et al. showed that the application of exosomal transfer into cells (EXOtic) devices for cell culture significantly increased exosome production and their therapeutic capability [69]. The EXOtic devices also enhanced the specific mRNA packaging and the delivery of the mRNA into the cytosol of the target cells, thus facilitating efficient cellular communication. The combination of biomaterials with cultivation technologies could also be used to further enhance exosome production.

#### 3.1.3. Downstream Modifications

To address the challenges associated with exosome isolation, new methods have been developed to improve exosome purity and achieve a greater yield. A one-step sucrose cushion ultracentrifugation was developed to improve the yield and purity of exosomes from the established ultracentrifugation. This procedure involves the addition of 30% sucrose solution followed by cell culture media, without mixing the layers [91]. Gupta et al. reported that the exosome cup-shaped morphology was greater than differential ultracentrifugation, thus demonstrating reduced size variability [91]. Modifications have also been made to other exosome isolation techniques, such as magnetic bead-based isolation and immunoaffinity chromatography. Smith et al. created a simple, size-based nanoscale deterministic lateral displacement array of microfluidic channels to collect exosomes, demonstrating ~50% recovery [92]. In a study by Z et al. an ExoSD microfluidic chip with an immunocapture-based method was developed to achieve exosome isolation [93]. The microfluidic chips reported >80% exosome recovery and >83% purity [93]. Heath et al. developed a cost-effective, high-throughput isolation technique called anion exchange chromatography to increase exosome yield [94]. Using higher flow rates and step elution, the authors utilized the net negative charge of exosomes to obtain 2.4x10^11^ exosomes, a quantity that was reported to be greater than that obtained using ultracentrifugation and tangential flow filtration [94]. 

Research into improving exosome isolation has also regarded the use of aptamer-based separation techniques [95,96]. Aptamers are single-stranded oligonucleotides that form distinct structures which bind to targets such as the extracellular components of exosomes (tetraspanins, transmembrane proteins). Zhang et al. developed a DNA aptamer-based magnetic isolation process to efficiently increase the yield of exosomes [97]. The process involved the addition of a biotin-labeled CD63 component to media and the subsequent separation of the labelled exosomes with streptavidin magnetic beads [97]. Another study by Song et al. also involved the use of a CD63-targeting aptamer for magnetic bead-based exosome immunoaffinity isolation [95]. Jiawei et al. developed a magnetic bead-based isolation technique in which tetraspanin markers (CD63, CD9, CD81) are combined with metal oxides for exosome isolation [24]. In addition, Zhang et al. discovered a novel three-step procedure involving PEG precipitation followed by iohexol gradient centrifugation and size exclusion chromatography for exosome enrichment and recovery [15]. Zhang et al. reported that the procedure produced high purity and yield of exosomes, resulting in 71% recovery and almost complete elimination of other lipoproteins [15]. Importantly, the modified or newly developed procedures for downstream modifications may assist in the large-scale use of exosomes as a drug delivery vehicle in a clinical setting.

### 3.2. Exosome Drug Loading

**Figure 3:**
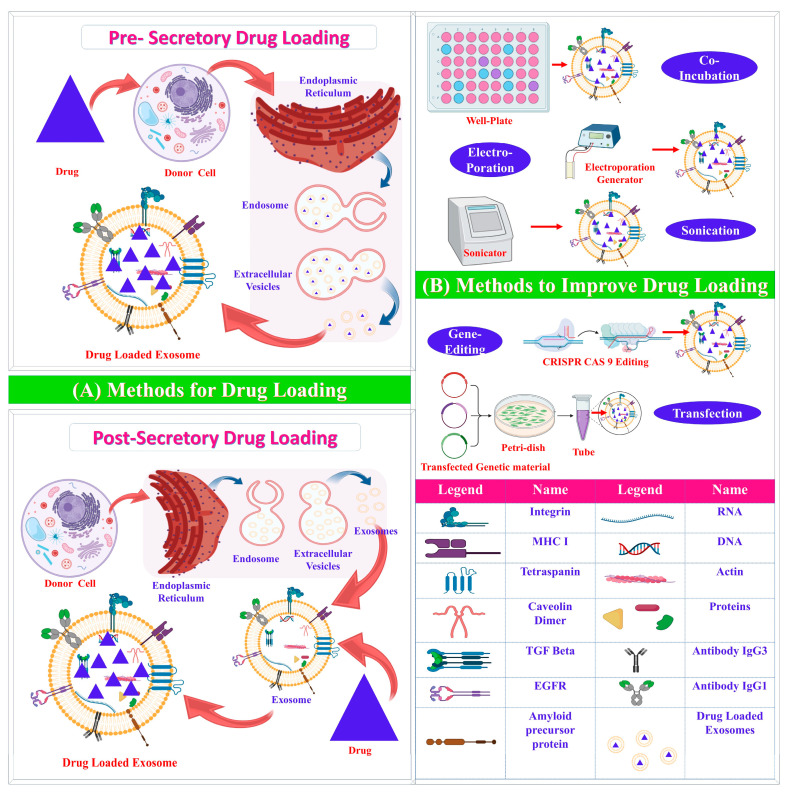
(**A**) Pre-secretory and post-secretory exosome drug-loading techniques and (**B**) methods of drug-loading enhancement. Pre-secretory drug loading, carried out before exosome secretion, involves (1) the incubation of the parent cell with the drug (transfection) and (2) gene editing. Post-secretory drug loading, carried out after exosome secretion, generally works in two ways: (1) passive incubation of the drug with the exosomes to allow the drug to attach to the exosome lipid bilayer membrane, or (2) the use of mechanical or chemical techniques, such as sonication and electroporation, to momentarily weaken the exosome membrane integrity to allow for the diffusion of the drug into the extracellular vesicles. The illustration was cre6ated with BioRender.com (https://app.biorender.com; accessed on 16 January 2023).

The clinical translation of exosomes requires reproducible and technologically accessible methods to load these extracellular vesicles with the desired drug (Figure 3). Several techniques have been modified or newly developed to assist in effective exosome drug loading, as discussed below.

#### 3.2.1. Pre-Secretory Drug Loading

Pre-secretory drug loading can be performed in two ways: (1) the incubation of a parent cell with the drug or (2) gene editing [98]. In incubation, the drug is directly mixed with the cell culture medium. The drug is internalized into the cells and subsequently loaded into exosomes via endogenous mechanisms. This technique is more effective in hydrophobic drugs due to their ability to interact with the exosome membrane. Research has shown that drugs such as methotrexate, doxorubicin, cisplatin, and paclitaxel have been successfully taken up by parent cells and loaded into exosomes for therapeutic treatment in different cancers [8,47,99]. Additionally, Zhang et al. demonstrated the transfection of parent cells with a siRNA-targeting tyrosine kinase c-Met in the treatment of gastric cancer [100]. The exosomes extracted, which were enriched with anti-c-Met siRNA, resulted in a significant decrease in tumor growth in mouse xenograft models, thus reversing the resistance of gastric cancer cells in vitro to cisplatin. Pre-secretory drug loading can also be accomplished through gene editing by adding plasmids to parent cells to produce exosomes enriched with nucleic acids or proteins. A study done by Yuan et al. demonstrated effective loading of the potent anti-cancer tumor necrosis factor-related apoptosis-induced ligand (TRAIL), a molecule known for its poor pharmaceutics, in mesenchymal stem cell-derived exosomes [30,101]. In addition, O’Brien et al. showed that miR-134 loaded exosomes were able to successfully reduce cellular invasion and migration and had improved sensitivity to anti-Hsp90 drugs [102,103]. Recently, a study by Yang et al. revealed that gene editing, coupled with nanoporation, successfully loaded a phosphatase and TENsin homolog deleted on chromosome 10 (PTEN) mRNA [104]. According to Yang et al., when loaded into exosomes, PTEN mRNA, a common tumor suppressor gene, produced a 50-fold increase in exosomes and a 1000-fold increase in exosomal mRNA transcripts compared to other drug-loading methods [104]. The authors further pointed out that large quantities of PTEN mRNA-containing exosomes were produced, and following systemic injection, displayed an increased survival rate in PTEN-deficient glioma mouse models [104]. Importantly, a novel pre-secretory drug-loading technique was developed by Nawaz et al. using lipid nanoparticles [105]. The authors delivered a therapeutic agent, VEGF-A mRNA, via lipid nanoparticles and studied the uptake kinetics and transport of the exogenous nanoparticles [105]. The results showed that the lipid nanoparticles altered the exosomes as functional extensions to distribute the therapeutic agent among cells [105]. Additionally, the exosomes themselves increased the production of the therapeutic component and other pro-angiogenesis agents for the treatment of inflammatory cardiac conditions [105]. Of note, the cell type used affected the functionality of the exosomes, whereby cardiac progenitor cells resulted in the lowest production of inflammatory agents [105]. The pre-secretory drug-loading method and the cell type used are important factors to consider for effective drug loading and the subsequent use of exosomes in a clinical setting.

#### 3.2.2. Post-Secretory Drug Loading

Post-secretory drug-loading methods generally work in two ways: (1) the passive incubation of the drug with the exosomes to allow the drug to attach to the exosome lipid bilayer membrane, or (2) the use of mechanical or chemical techniques to momentarily weaken the integrity of the exosome membrane to allow for the diffusion of the drug into the extracellular vesicles. With the increased interest in the use of exosomes as drug delivery tools, new approaches for post-secretory drug loading have been considered over the last few years. Wang et al. developed an acoustofluidic device, which is a combination of fluid mechanics and acoustics, to perform both exosome drug loading and encapsulation with silica nanoparticles [39,106,107,108]. In this single-step process, drug loading significantly improved with a reported 70% efficacy [39]. 

Methods based on liposome–exosome fusion have also recently been proposed [109,110]. Additionally, Li et al. successfully incubated and merged the cargo of exosomes with liposomes containing fusogenic lipids, providing an alternative approach to the efficient loading of larger molecules [110]. Liposome–exosome hybrids allow for the incorporation of drugs without compromising the exosome membrane. It combines the advantages of the liposomes (ease of drug loading) with that of the exosomes (biocompatibility and targeting abilities) for effective drug loading and delivery. In another study, Yim et al. established a unique optogenetic exosome system via optically reversible protein–protein interactions (EXPLORs) [48]. The effective loading of cargo proteins into the exosomes was demonstrated using a reversible protein–protein interaction module controlled by blue light via the exosome endogenous biogenesis pathway [48]. It was noted that the protein-loaded EXPLORs delivered to the cytosols of target cells resulted in a significant increase in the intracellular levels of cargo proteins and their functions in vitro and in vivo [48]. Osteikoetxea et al. developed a new method for the successful loading of the clustered regularly interspaced short palindromic repeats (CRISPR/Cas9) into exosomes through the reversible heterodimerization of Cas9 fusions with exosome-specific components, such as tetraspanins [111]. 

New drug-loading methods have also considered the use of ubiquitination tags as a sorting sequence to facilitate effective drug loading [112]. An engineered ubiquitin tag was developed, and its fusion with proteins, such as enhanced green fluorescent protein, led to the loading of proteins into the exosome [112]. Another method involving a short ubiquitin tag with specific binding to the L-domain motif of Ndfip1 resulted in the efficient loading of proteins into exosomes [32]. The use of a non-functional mutant Nef protein facilitated the sorting of proteins into exosomes through its association with the exosomal lipid–raft microdomains [112]. In addition, Sutaria et al. developed a mechanism for the effective loading of miR-199a into exosomes via the trans-activating response element sequence, trans-activator of transcription, and Lamp2a (a component responsible for the loading of proteins into exosomes) [113]. In one study, HuR, an RNA-binding protein, was fused to the tetraspanin CD9 to be localized in the exosomal lumen to facilitate the loading of miR-155 into the exosome [114]. These alternative drug-loading methods, coupled with exosome isolation methods, may assist in the large-scale use of exosomes in a clinical setting.

### 3.3. Targeted Exosome Delivery

In addition to effective drug loading, the development of targeted exosomes that are capable of high specificity and prolonged therapeutic function is of importance, as the ability of exosomes to administer the therapeutics to specific organs/tissues would reduce the possibility of undesired cellular interactions. Moreover, the administration of targeted exosomes would result in prolonged systemic circulation through the evasion of the mononuclear phagocyte system, which would aid in improving the therapeutic value of exosomes in nanomedicine. As such, a study incorporated various techniques to develop effective targeted exosomes and protection from the mononuclear phagocyte system [30]. The most common approach involves the grafting of hydrophilic polymers, such as polyethylene glycol (PEG), onto the exosome lipid bilayer membrane. The contact between exosomes and opsonin is impeded by these hydrophilic polymers, thus leading to prolonged systemic circulation. To circumvent this, Antes et al. engineered a protective ‘cloaking’ platform for modified exosomes to reduce their clearance by the phagocyte system [115]. However, despite its simplicity, cloaking must be done on each exosome, and as such, can be time-consuming. 

Another approach to the development of targeted exosomes involves the modification of the glycan composition of the surface of exosomes, which plays an important role in uptake and cellular recognition [116]. Royo et al. reported that changes made to the sialic residues from glycoproteins produced targeted exosomes for specific organs [117]. Guo et al. developed targeted exosomes for bone tissue by the insertion of Golgi glycoprotein 1 into the exosome membrane [118]. The glycoprotein carried Wnt agonist 1, which reportedly reduced bone loss, accelerated fracture healing in colitis, and increased bone formation in mice [118]. Moreover, the presence of negatively charged phospholipids on exosomes increased their clearance through macrophages. Accordingly, research involving the blocking of the phospholipids has resulted in the prolonged circulation of exosomes.

Other approaches to improve the targeting ability of exosomes include the alterations of integrins and the use of aptamers [4]. The different integrins located on the surface of exosomes affect their pharmacokinetics and can be used to increase the accumulation of exosomes in tissues. Rana et al. were able to increase the selective uptake of exosomes in pancreatic cells by combining the protein Tspan with the extracellular exosome component integrin α4 [119]. In addition to their use in exosome isolation, aptamers have also been shown to improve the targeting ability of exosomes. Research by Zou et al. developed aptamer-functionalized exosomes for cell-type-specific delivery of therapeutics [120]. The recognition capability of aptamers and the transport functions of exosomes were combined to effectively deliver molecular therapeutics or fluorophores to target tumor cells [120]. 

The incorporation of targeted exosomes with various drug-loading methods can increase the therapeutic value and efficacy of exosomes as a drug delivery tool. Liang et al. targeted colon cells specifically by fusing Her-2 to the N-terminus of Lamp2 on exosomes [121]. Following the alteration of the exosome membrane, two therapeutics—5-fluorouracil (electroporation) and miRNA-21 inhibitor (incubation)—were incorporated into the exosomes [121]. The authors noted that the method enhanced cellular uptake via the EGFR receptor-mediated endocytosis in colon cancer cells and successfully suppressed the tumor [121]. A study by Xu et al. demonstrated the specificity of kartogenin-loaded-targeted exosomes to the synovial fluid-derived mesenchymal stem cells by the addition of a specific mesenchymal stem cell-binding peptide (E7) to the exosome surface. The peptide was bound to Lamp2b, found on the surface of exosomes, and promoted mesenchymal stem cell chondrogenic differentiation and cartilage repair [122]. In a study by Jia et al., exosomes were loaded with superparamagnetic iron oxide nanoparticles and curcumin, followed by the conjugation of the exosome membrane with neuropilin-1-targeted peptides using click chemistry [123]. Through imaging and therapeutic analysis, Jia et al. reported the successful production of glioma-targeting exosomes [123]. Targeted exosomes in combination with drug-loading mechanisms are invaluable to the effective use of these extracellular vesicles as a drug delivery tool in the therapeutic treatment of various diseases in a large-scale clinical environment. 

## 4. Conclusions

Exosomes are nanosized lipid-based extracellular vesicles that play an important role in cellular communication and cargo transport. The immunomodulatory, pharmacokinetic, and biocompatibility ability of exosomes have rendered these extracellular vesicles invaluable as a therapeutic approach for countless diseases. Studies involving the implementation of exosome-based therapies in the treatment of various diseases have shown great promise. However, the use of exosome-based therapies in clinical settings is hindered by several challenges that require attention. One of the most significant challenges is exosome production and isolation. Importantly, exosome drug loading has proven difficult, as its effectiveness depends on the type of drug to be loaded and the source of the exosome. However, as the interest in exosomes as potential therapeutic agents grows, new mechanisms and modifications have been made to improve exosome isolation and drug loading for their possible use in nanomedicine.

## Figures and Tables

**Table 1:** Comparison of different downstream exosome isolation techniques. The table summarizes the advantages and disadvantages of each technique and their reported exosome recovery rate.

Isolation Technique	Principle	Recovery (%)	Pros	Cons	References
Ultracentrifugation	Sedimentation rate	5–20	High sample capacity and low cost	Time-consuming and low purity	[9,10]
Density gradient ultracentrifugation	Density, size, shape	10–40	High purity and protein concentration	Long run time and low yield	[9,11,12]
Polymer-based precipitation	Sedimentation rate	90+	High yield	Low purity	[13,14,15]
Ultrafiltration	Size	30	Maintains integrity; simple and low-cost	Moderate purity; low yield due to exosome trapping in filter pores	[9,16,17]
Size-exclusion chromatography	Size	40–80	High purity, integrity, and functionality; reduction of exosome aggregation	Low extraction volume	[9,18]
Immunoaffinity chromatography	Surface marker	90+	Maintain integrity	Low capacity and low yield	[9,19,20]
Microfluidics	Surface marker	40–90	Low cost and low input sample required	Low sample capacity; cargo may be modified	[9,21,22]
Magnetic bead isolation	Surface marker	80+	Maintain integrity	Possible impurities	[23,24]

**Table 2:** A list of the different exosome drug-loading techniques and their advantages and disadvantages.

Methods	Principle	Advantages	Disadvantages	References
**Pre-secretory Drug Loading**				
Co-incubation	Drug incubated with parent cell	Easy; effective in hydrophobic drugs	Low loading efficacy; possible drug toxicity	[31]
Gene editing	Editing of genes	Overexpression of specific molecules	Low loading efficacy; possible toxicity	[32]
**Post-Secretory Drug Loading**				
Sonication	Mechanical shear force decreases membrane integrity	Large amount of drug loaded	Possible damage to intracellular components and integrity	[3,33,34]
Electroporation	High-voltage electric charge decreases membrane integrity	Effective loading of hydrophilic drugs and nucleic acids	Possible aggregation; low loading efficacy	[35]
Passive incubation	Passive diffusion	Effective loading of hydrophobic drugs; does not affect exosome integrity	Not useful for hydrophilic drugs; low drug-loading capacity	[3,34,36,37,38,39]
Freeze–thaw	Repeated freeze–thaw cycles to decrease membrane integrity	Easy process	Low loading efficacy; possible aggregation and inactivation	[3,40]
Nanoporation	Nanosecond electrical pulse decreases membrane integrity	Effective loading of small molecules	Possible aggregation	[41,42]
Saponin treatment	Formation of porous structure on exosome membrane	Increased loading capacity compared to electroporation	May cause hemolysis in vivo; requires further purification	[3,43]
Extrusion	Mechanical stress decreases membrane integrity	Provides uniform distribution	May damage membrane; possible drug leakage	[3,44]

**Table 3:** Comparison of different upstream modifications for increased exosome production and their reported fold increase and effects.

Upstream Modifications	Fold Increase	Alterations and Effects	References
**Soluble Factors**			
Lipopolysaccharide (LPS)	1.37	Upregulation of let-7b increased immunotherapeutic effect	[62]
N-methyldopamine and norepinephrine	3	No significant change	[63]
Serotonin and calcium	2–2.5	-	[64]
Adiponectin	3	Present in exosomes	[65]
ATP	4	No significant change	[66]
Wnt3a	-	Present in exosomes; increased neuroprotective abilities	[67]
Plant ceramide	2.5	-	[68]
**Chemical/physical stimulation**			
Hypoxia	1.5	Dependent on cell type; increased expression of nucleic acids and proteins	[71,72,74,75]
Serum deprivation	Varies	Decreased exosome protein content	[52,76]
Flow/stretch	37	Over 200 proteins expressed differently from typical exosomes	[77,78]
High-frequency ultrasound	8–10	Increased exosome protein content	[79]
**3D cultivation**			
3D spheroid culture	2–3	-	[80]
Microcarrier-based suspension	20; 140 with tangential flow system	No significant change	[52,81,82,83]
3D print fibrillar scaffold with perfusion system	100	Decreased exosome protein content	[84]
Low-shear unsubmerged 3D-printed polylactic acid lattice matrix	2	Maintained protein expression	[85]
**Biomaterials**			
Nitric oxide-releasing polymer	Not significant	Enhanced pro-angiogenic activity	[86]
Lithium-incorporated bioactive glass ceramic	Not significant	Enhanced pro-angiogenic activity	[87]
Iron-oxide coated poly-lactic-co-glycosidic acid (PLGA) nanoparticle	2	Increased antioxidant or tissue regeneration factors	[88]
Bioglass	2	Modulation of cargo through altered expression of microRNA; enhanced ability to promote vascularization	[89]
EXOtic	~6.8	-	[69]

## Data Availability

Data sharing not applicable.

## Abbreviations

CRISPR/CAS9	Clustered regularly interspaced short palindromic repeats-associated protein 9
ESCRT	Endosomal sorting complex required for transport
ESE	Early-sorting endosome
EXOSD	Exosome separation and detection
EXOtic	Exosomal transfer into cells
EXPLOR	Exosome system via optically reversible protein–protein interactions
HIFα	Hypoxia-induced factor α
HUR	Human antigen R
ILV	Intraluminal vesicle
LPS	Lipopolysaccharide
LSE	Late-sorting endosome
MVB	Multivesicular body
NDFIP1	Nedd4 family interacting protein 1
NEF	Negative regulatory factor
PEG	Polyethylene glycol
PLGA	Poly(lactic-co-glycolic acid)
PTEN	Phosphatase and TENsin homolog deleted on chromosome 10
STEAP3	Six-transmembrane epithelial antigen of prostate 3
TRAIL	Tumor necrosis factor-related apoptosis-induced ligand

## References

[1] Ha D., Yang N., Nadithe V. (2016). Exosomes as Therapeutic Drug Carriers and Delivery Vehicles across Biological Membranes: Current Perspectives and Future Challenges. Acta Pharm. Sin. B.

[2] Zhang Y., Bi J., Huang J., Tang Y., Du S., Li P. (2020). Exosome: A Review of Its Classification, Isolation Techniques, Storage, Diagnostic and Targeted Therapy Applications. Int. J. Nanomed..

[3] Haney M.J., Klyachko N.L., Zhao Y., Gupta R., Plotnikova E.G., He Z., Patel T., Piroyan A., Sokolsky M., Kabanov A.V. (2015). Exosomes as Drug Delivery Vehicles for Parkinson’s Disease Therapy. J. Control. Release.

[4] Elsharkasy O.M., Nordin J.Z., Hagey D.W., de Jong O.G., Schiffelers R.M., Andaloussi S.E., Vader P. (2020). Extracellular Vesicles as Drug Delivery Systems: Why and How?. Adv. Drug Deliv. Rev..

[5] An Y., Lin S., Tan X., Zhu S., Nie F., Zhen Y., Gu L., Zhang C., Wang B., Wei W. (2021). Exosomes from Adipose-derived Stem Cells and Application to Skin Wound Healing. Cell Prolif..

[6] Kumar D.N., Chaudhuri A., Aqil F., Dehari D., Munagala R., Singh S., Gupta R.C., Agrawal A.K. (2022). Exosomes as Emerging Drug Delivery and Diagnostic Modality for Breast Cancer: Recent Advances in Isolation and Application. Cancers.

[7] Hussen B.M., Faraj G.S.H., Rasul M.F., Hidayat H.J., Salihi A., Baniahmad A., Taheri M., Ghafouri-Frad S. (2022). Strategies to Overcome the Main Challenges of the Use of Exosomes as Drug Carrier for Cancer Therapy. Cancer Cell Int..

[8] Zhang Y., Li J., Gao W., Xie N. (2022). Exosomes as Anticancer Drug Delivery Vehicles: Prospects and Challenges. Front. Biosci.-Landmark.

[9] Ramirez M.I., Amorim M.G., Gadelha C., Milic I., Welsh J.A., Freitas V.M., Nawaz M., Akbar N., Couch Y., Makin L. (2018). Technical Challenges of Working with Extracellular Vesicles. Nanoscale.

[10] Livshits M.A., Khomyakova E., Evtushenko E.G., Lazarev V.N., Kulemin N.A., Semina S.E., Generozov E.V., Govorun V.M. (2015). Isolation of Exosomes by Differential Centrifugation: Theoretical Analysis of a Commonly Used Protocol. Sci. Rep..

[11] Li K., Wong D.K., Hong K.Y., Raffai R.L. (2018). Cushioned-Density Gradient Ultracentrifugation (C-DGUC): A Refined and High Performance Method for the Isolation, Characterization, and Use of Exosomes. Methods Mol. Biol..

[12] Onódi Z., Pelyhe C., Terézia Nagy C., Brenner G.B., Almási L., Kittel Á., Manček-Keber M., Ferdinandy P., Buzás E.I., Giricz Z. (2018). Isolation of High-Purity Extracellular Vesicles by the Combination of Iodixanol Density Gradient Ultracentrifugation and Bind-Elute Chromatography from Blood Plasma. Front. Physiol..

[13] Rider M.A., Hurwitz S.N., Meckes D.G. (2016). ExtraPEG: A Polyethylene Glycol-Based Method for Enrichment of Extracellular Vesicles. Sci. Rep..

[14] Emam S.E., Ando H., Lila A.S.A., Shimizu T., Ukawa M., Okuhira K., Ishima Y., Mahdy M.A., Ghazy F.S., Ishida T. (2018). A Novel Strategy to Increase the Yield of Exosomes (Extracellular Vesicles) for an Expansion of Basic Research. Biol. Pharm. Bull..

[15] Zhang X., Borg E.G.F., Liaci A.M., Vos H.R., Stoorvogel W. (2020). A Novel Three Step Protocol to Isolate Extracellular Vesicles from Plasma or Cell Culture Medium with Both High Yield and Purity. J. Extracell. Vesicles.

[16] Benedikter B.J., Bouwman F.G., Vajen T., Heinzmann A.C.A., Grauls G., Mariman E.C., Wouters E.F.M., Savelkoul P.H., Lopez-Iglesias C., Koenen R.R. (2017). Ultrafiltration Combined with Size Exclusion Chromatography Efficiently Isolates Extracellular Vesicles from Cell Culture Media for Compositional and Functional Studies. Sci. Rep..

[17] Guerreiro E.M., Vestad B., Steffensen L.A., Aass H.C.D., Saeed M., Øvstebø R., Costea D.E., Galtung H.K., Søland T.M. (2018). Efficient Extracellular Vesicle Isolation by Combining Cell Media Modifications, Ultrafiltration, and Size-Exclusion Chromatography. PLoS ONE.

[18] Gámez-Valero A., Monguió-Tortajada M., Carreras-Planella L., Franquesa M., Beyer K., Borràs F.E. (2016). Size-Exclusion Chromatography-Based Isolation Minimally Alters Extracellular Vesicles’ Characteristics Compared to Precipitating Agents. Sci. Rep..

[19] Sharma P., Ludwig S., Muller L., Hong C.S., Kirkwood J.M., Ferrone S., Whiteside T.L. (2018). Immunoaffinity-Based Isolation of Melanoma Cell-Derived Exosomes from Plasma of Patients with Melanoma. J. Extracell. Vesicles.

[20] Filipović L., Spasojević M., Prodanović R., Korać A., Matijaševic S., Brajušković G., de Marco A., Popović M. (2022). Affinity-Based Isolation of Extracellular Vesicles by Means of Single-Domain Antibodies Bound to Macroporous Methacrylate-Based Copolymer. New Biotechnol..

[21] Contreras-Naranjo J.C., Wu H.-J., Ugaz V.M. (2017). Microfluidics for Exosome Isolation and Analysis: Enabling Liquid Biopsy for Personalized Medicine. Lab Chip.

[22] Talebjedi B., Tasnim N., Hoorfar M., Mastromonaco G.F., De Almeida Monteiro Melo Ferraz M. (2021). Exploiting Microfluidics for Extracellular Vesicle Isolation and Characterization: Potential Use for Standardized Embryo Quality Assessment. Front. Vet. Sci..

[23] Sun J., Han S., Ma L., Zhang H., Zhan Z., Aguilar H.A., Zhang H., Xiao K., Gu Y., Gu Z. (2021). Synergistically Bifunctional Paramagnetic Separation Enables Efficient Isolation of Urine Extracellular Vesicles and Downstream Phosphoproteomic Analysis. ACS Appl. Mater. Interfaces.

[24] Jiawei S., Zhi C., Kewei T., Xiaoping L. (2022). Magnetic Bead-Based Adsorption Strategy for Exosome Isolation. Front. Bioeng. Biotechnol..

[25] Paolini L., Monguió-Tortajada M., Costa M., Antenucci F., Barilani M., Clos-Sansalvador M., Andrade A.C., Driedonks T.A.P., Giancaterino S., Kronstadt S.M. (2022). Large-Scale Production of Extracellular Vesicles: Report on the “MassivEVs” ISEV Workshop. J. Extracell. Biol..

[26] Jeyaram A., Jay S.M. (2017). Preservation and Storage Stability of Extracellular Vesicles for Therapeutic Applications. AAPS J..

[27] Lőrincz Á.M., Timár C.I., Marosvári K.A., Veres D.S., Otrokocsi L., Kittel Á., Ligeti E. (2014). Effect of Storage on Physical and Functional Properties of Extracellular Vesicles Derived from Neutrophilic Granulocytes. J. Extracell. Vesicles.

[28] Maroto R., Zhao Y., Jamaluddin M., Popov V.L., Wang H., Kalubowilage M., Zhang Y., Luisi J., Sun H., Culbertson C.T. (2017). Effects of Storage Temperature on Airway Exosome Integrity for Diagnostic and Functional Analyses. J. Extracell. Vesicles.

[29] Bunggulawa E.J., Wang W., Yin T., Wang N., Durkan C., Wang Y., Wang G. (2018). Recent Advancements in the Use of Exosomes as Drug Delivery Systems. J. Nanobiotechnol..

[30] Ferreira D., Moreira J.N., Rodrigues L.R. (2022). New Advances in Exosome-Based Targeted Drug Delivery Systems. Crit. Rev. Oncol./Hematol..

[31] Pascucci L., Coccè V., Bonomi A., Ami D., Ceccarelli P., Ciusani E., Viganò L., Locatelli A., Sisto F., Doglia S.M. (2014). Paclitaxel Is Incorporated by Mesenchymal Stromal Cells and Released in Exosomes That Inhibit in vitro Tumor Growth: A New Approach for Drug Delivery. J. Control. Release.

[32] Sterzenbach U., Putz U., Low L.-H., Silke J., Tan S.-S., Howitt J. (2017). Engineered Exosomes as Vehicles for Biologically Active Proteins. Mol. Ther..

[33] Liu C., Zhang W., Li Y., Chang J., Tian F., Zhao F., Ma Y., Sun J. (2019). Microfluidic Sonication to Assemble Exosome Membrane-Coated Nanoparticles for Immune Evasion-Mediated Targeting. Nano Lett..

[34] Salarpour S., Forootanfar H., Pournamdari M., Ahmadi-Zeidabadi M., Esmaeeli M., Pardakhty A. (2019). Paclitaxel Incorporated Exosomes Derived from Glioblastoma Cells: Comparative Study of Two Loading Techniques. Daru.

[35] Rodríguez-Morales B., Antunes-Ricardo M., González-Valdez J. (2021). Exosome-Mediated Insulin Delivery for the Potential Treatment of Diabetes Mellitus. Pharmaceutics.

[36] Tang M., Chen Y., Li B., Sugimoto H., Yang S., Yang C., LeBleu V.S., McAndrews K.M., Kalluri R. (2021). Therapeutic Targeting of STAT3 with Small Interference RNAs and Antisense Oligonucleotides Embedded Exosomes in Liver Fibrosis. FASEB J..

[37] Deng W., Meng Y., Wang B., Wang C.-X., Hou C.-X., Zhu Q.-H., Tang Y.-T., Ye J.-H. (2022). In Vitro Experimental Study on the Formation of MicroRNA-34a Loaded Exosomes and Their Inhibitory Effect in Oral Squamous Cell Carcinoma. Cell Cycle.

[38] González-Sarrías A., Iglesias-Aguirre C.E., Cortés-Martín A., Vallejo F., Cattivelli A., del Pozo-Acebo L., Del Saz A., López de las Hazas M.C., Dávalos A., Espín J.C. (2022). Milk-Derived Exosomes as Nanocarriers to Deliver Curcumin and Resveratrol in Breast Tissue and Enhance Their Anticancer Activity. Int. J. Mol. Sci..

[39] Wang Z., Rich J., Hao N., Gu Y., Chen C., Yang S., Zhang P., Huang T.J. (2022). Acoustofluidics for Simultaneous Nanoparticle-Based Drug Loading and Exosome Encapsulation. Microsyst. Nanoeng..

[40] Sato Y.T., Umezaki K., Sawada S., Mukai S., Sasaki Y., Harada N., Shiku H., Akiyoshi K. (2016). Engineering Hybrid Exosomes by Membrane Fusion with Liposomes. Sci. Rep..

[41] Yang X., Shi G., Guo J., Wang C., He Y. (2018). Exosome-Encapsulated Antibiotic against Intracellular Infections of Methicillin-Resistant Staphylococcus Aureus. Int. J. Nanomed..

[42] Hao R., Yu Z., Du J., Hu S., Yuan C., Guo H., Zhang Y., Yang H. (2021). A High-Throughput Nanofluidic Device for Exosome Nanoporation to Develop Cargo Delivery Vehicles. Small.

[43] Fuhrmann G., Chandrawati R., Parmar P.A., Keane T.J., Maynard S.A., Bertazzo S., Stevens M.M. (2018). Engineering Extracellular Vesicles with the Tools of Enzyme Prodrug Therapy. Adv. Mater..

[44] Fan J., Lee C.-S., Kim S., Chen C., Aghaloo T., Lee M. (2020). Generation of Small RNA-Modulated Exosome Mimetics for Bone Regeneration. ACS Nano.

[45] McAndrews K.M., Che S.P.Y., LeBleu V.S., Kalluri R. (2021). Effective Delivery of STING Agonist Using Exosomes Suppresses Tumor Growth and Enhances Antitumor Immunity. J. Biol. Chem..

[46] Wei H., Chen J., Wang S., Fu F., Zhu X., Wu C., Liu Z., Zhong G., Lin J. (2019). A Nanodrug Consisting of Doxorubicin and Exosome Derived from Mesenchymal Stem Cells for Osteosarcoma Treatment in vitro. Int. J. Nanomed..

[47] Yang T., Martin P., Fogarty B., Brown A., Schurman K., Phipps R., Yin V.P., Lockman P., Bai S. (2015). Exosome Delivered Anticancer Drugs Across the Blood-Brain Barrier for Brain Cancer Therapy in Danio Rerio. Pharm. Res..

[48] Yim N., Ryu S.-W., Choi K., Lee K.R., Lee S., Choi H., Kim J., Shaker M.R., Sun W., Park J.-H. (2016). Exosome Engineering for Efficient Intracellular Delivery of Soluble Proteins Using Optically Reversible Protein–Protein Interaction Module. Nat. Commun..

[49] Burrello J., Monticone S., Gai C., Gomez Y., Kholia S., Camussi G. (2016). Stem Cell-Derived Extracellular Vesicles and Immune-Modulation. Front. Cell Dev. Biol..

[50] Ishiy C.S.R.A., Ormanji M.S., Maquigussa E., Ribeiro R.S., da Silva Novaes A., Boim M.A. (2020). Comparison of the Effects of Mesenchymal Stem Cells with Their Extracellular Vesicles on the Treatment of Kidney Damage Induced by Chronic Renal Artery Stenosis. Stem. Cells Int..

[51] Fernández-Francos S., Eiro N., Costa L.A., Escudero-Cernuda S., Fernández-Sánchez M.L., Vizoso F.J. (2021). Mesenchymal Stem Cells as a Cornerstone in a Galaxy of Intercellular Signals: Basis for a New Era of Medicine. Int. J. Mol. Sci..

[52] Haraszti R.A., Miller R., Stoppato M., Sere Y.Y., Coles A., Didiot M.-C., Wollacott R., Sapp E., Dubuke M.L., Li X. (2018). Exosomes Produced from 3D Cultures of MSCs by Tangential Flow Filtration Show Higher Yield and Improved Activity. Mol. Ther..

[53] Schwechheimer C., Kuehn M.J. (2015). Outer-Membrane Vesicles from Gram-Negative Bacteria: Biogenesis and Functions. Nat. Rev. Microbiol..

[54] Liu Y., Defourny K.A.Y., Smid E.J., Abee T. (2018). Gram-Positive Bacterial Extracellular Vesicles and Their Impact on Health and Disease. Front. Microbiol..

[55] Bitto N.J., Zavan L., Johnston E.L., Stinear T.P., Hill A.F., Kaparakis-Liaskos M. (2021). Considerations for the Analysis of Bacterial Membrane Vesicles: Methods of Vesicle Production and Quantification Can Influence Biological and Experimental Outcomes. Microbiol. Spectr..

[56] Agrawal A.K., Aqil F., Jeyabalan J., Spencer W.A., Beck J., Gachuki B.W., Alhakeem S.S., Oben K., Munagala R., Bondada S. (2017). Milk-Derived Exosomes for Oral Delivery of Paclitaxel. Nanomedicine.

[57] Marcilla A., Trelis M., Cortés A., Sotillo J., Cantalapiedra F., Minguez M.T., Valero M.L., del Pino M.M.S., Muñoz-Antoli C., Toledo R. (2012). Extracellular Vesicles from Parasitic Helminths Contain Specific Excretory/Secretory Proteins and Are Internalized in Intestinal Host Cells. PLoS ONE.

[58] Teng Y., Ren Y., Sayed M., Hu X., Lei C., Kumar A., Hutchins E., Mu J., Deng Z., Luo C. (2018). Plant-Derived Exosomal MicroRNAs Shape the Gut Microbiota. Cell Host. Microbe.

[59] Adamo G., Fierli D., Romancino D.P., Picciotto S., Barone M.E., Aranyos A., Božič D., Morsbach S., Raccosta S., Stanly C. (2021). Nanoalgosomes: Introducing Extracellular Vesicles Produced by Microalgae. J. Extracell. Vesicles.

[60] Picciotto S., Barone M.E., Fierli D., Aranyos A., Adamo G., Božič D., Romancino D.P., Stanly C., Parkes R., Morsbach S. (2021). Isolation of Extracellular Vesicles from Microalgae: Towards the Production of Sustainable and Natural Nanocarriers of Bioactive Compounds. Biomater. Sci..

[61] Petousis-Harris H. (2018). Impact of Meningococcal Group B OMV Vaccines, beyond Their Brief. Hum. Vaccines Immunother..

[62] Ti D., Hao H., Tong C., Liu J., Dong L., Zheng J., Zhao Y., Liu H., Fu X., Han W. (2015). LPS-Preconditioned Mesenchymal Stromal Cells Modify Macrophage Polarization for Resolution of Chronic Inflammation via Exosome-Shuttled Let-7b. J. Transl. Med..

[63] Wang J., Bonacquisti E.E., Brown A.D., Nguyen J. (2020). Boosting the Biogenesis and Secretion of Mesenchymal Stem Cell-Derived Exosomes. Cells.

[64] Glebov K., Löchner M., Jabs R., Lau T., Merkel O., Schloss P., Steinhäuser C., Walter J. (2015). Serotonin Stimulates Secretion of Exosomes from Microglia Cells: Serotonin Stimulates Microglial Exosome Release. Glia.

[65] Nakamura Y., Kita S., Tanaka Y., Fukuda S., Obata Y., Okita T., Nishida H., Takahashi Y., Kawachi Y., Tsugawa-Shimizu Y. (2020). Adiponectin Stimulates Exosome Release to Enhance Mesenchymal Stem-Cell-Driven Therapy of Heart Failure in Mice. Mol. Ther..

[66] Drago F., Lombardi M., Prada I., Gabrielli M., Joshi P., Cojoc D., Franck J., Fournier I., Vizioli J., Verderio C. (2017). ATP Modifies the Proteome of Extracellular Vesicles Released by Microglia and Influences Their Action on Astrocytes. Front. Pharmacol..

[67] Hooper C., Sainz-Fuertes R., Lynham S., Hye A., Killick R., Warley A., Bolondi C., Pocock J., Lovestone S. (2012). Wnt3a Induces Exosome Secretion from Primary Cultured Rat Microglia. BMC Neurosci..

[68] Yuyama K., Takahashi K., Usuki S., Mikami D., Sun H., Hanamatsu H., Furukawa J., Mukai K., Igarashi Y. (2019). Plant Sphingolipids Promote Extracellular Vesicle Release and Alleviate Amyloid-β Pathologies in a Mouse Model of Alzheimer’s Disease. Sci. Rep..

[69] Kojima R., Bojar D., Rizzi G., Hamri G.C.-E., El-Baba M.D., Saxena P., Ausländer S., Tan K.R., Fussenegger M. (2018). Designer Exosomes Produced by Implanted Cells Intracerebrally Deliver Therapeutic Cargo for Parkinson’s Disease Treatment. Nat. Commun..

[70] Li X., Corbett A.L., Taatizadeh E., Tasnim N., Little J.P., Garnis C., Daugaard M., Guns E., Hoorfar M., Li I.T.S. (2019). Challenges and Opportunities in Exosome Research—Perspectives from Biology, Engineering, and Cancer Therapy. APL Bioeng..

[71] Gonzalez-King H., García N.A., Ontoria-Oviedo I., Ciria M., Montero J.A., Sepúlveda P. (2017). Hypoxia Inducible Factor-1α Potentiates Jagged 1-Mediated Angiogenesis by Mesenchymal Stem Cell-Derived Exosomes. Stem Cells.

[72] Zhang W., Zhou X., Yao Q., Liu Y., Zhang H., Dong Z. (2017). HIF-1-Mediated Production of Exosomes during Hypoxia Is Protective in Renal Tubular Cells. Am. J. Physiol. Ren. Physiol..

[73] Böker K.O., Lemus-Diaz N., Ferreira R.R., Schiller L., Schneider S., Gruber J. (2018). The Impact of the CD9 Tetraspanin on Lentivirus Infectivity and Exosome Secretion. Mol. Ther..

[74] Cui G.-H., Wu J., Mou F.-F., Xie W.-H., Wang F.-B., Wang Q.-L., Fang J., Xu Y.-W., Dong Y.-R., Liu J.-R. (2018). Exosomes Derived from Hypoxia-Preconditioned Mesenchymal Stromal Cells Ameliorate Cognitive Decline by Rescuing Synaptic Dysfunction and Regulating Inflammatory Responses in APP/PS1 Mice. FASEB J..

[75] Jiang H., Zhao H., Zhang M., He Y., Li X., Xu Y., Liu X. (2022). Hypoxia Induced Changes of Exosome Cargo and Subsequent Biological Effects. Front. Immunol..

[76] Garcia N.A., Ontoria-Oviedo I., González-King H., Diez-Juan A., Sepúlveda P. (2015). Glucose Starvation in Cardiomyocytes Enhances Exosome Secretion and Promotes Angiogenesis in Endothelial Cells. PLoS ONE.

[77] Deng Z., Wang J., Xiao Y., Li F., Niu L., Liu X., Meng L., Zheng H. (2021). Ultrasound-Mediated Augmented Exosome Release from Astrocytes Alleviates Amyloid-β-Induced Neurotoxicity. Theranostics.

[78] Guo Y., Wan Z., Zhao P., Wei M., Liu Y., Bu T., Sun W., Li Z., Yuan L. (2021). Ultrasound Triggered Topical Delivery of Bmp7 MRNA for White Fat Browning Induction via Engineered Smart Exosomes. J. Nanobiotechnol..

[79] Ambattu L.A., Ramesan S., Dekiwadia C., Hanssen E., Li H., Yeo L.Y. (2020). High Frequency Acoustic Cell Stimulation Promotes Exosome Generation Regulated by a Calcium-Dependent Mechanism. Commun. Biol..

[80] Kim M., Yun H.-W., Park D.Y., Choi B.H., Min B.-H. (2018). Three-Dimensional Spheroid Culture Increases Exosome Secretion from Mesenchymal Stem Cells. Tissue Eng. Regen. Med..

[81] Cao J., Wang B., Tang T., Lv L., Ding Z., Li Z., Hu R., Wei Q., Shen A., Fu Y. (2020). Three-Dimensional Culture of MSCs Produces Exosomes with Improved Yield and Enhanced Therapeutic Efficacy for Cisplatin-Induced Acute Kidney Injury. Stem Cell Res. Ther..

[82] Koh B., Sulaiman N., Fauzi M.B., Law J.X., Ng M.H., Idrus R.B.H., Yazid M.D. (2020). Three Dimensional Microcarrier System in Mesenchymal Stem Cell Culture: A Systematic Review. Cell Biosci..

[83] Xu C., Zhao J., Li Q., Hou L., Wang Y., Li S., Jiang F., Zhu Z., Tian L. (2020). Exosomes Derived from Three-Dimensional Cultured Human Umbilical Cord Mesenchymal Stem Cells Ameliorate Pulmonary Fibrosis in a Mouse Silicosis Model. Stem Cell Res. Ther..

[84] Patel D.B., Luthers C.R., Lerman M.J., Fisher J.P., Jay S.M. (2019). Enhanced Extracellular Vesicle Production and Ethanol-Mediated Vascularization Bioactivity via a 3D-Printed Scaffold-Perfusion Bioreactor System. Acta Biomater..

[85] Burns A.B., Doris C., Vehar K., Saxena V., Bardliving C., Shamlou P.A., Phillips M.I. (2021). Novel Low Shear 3D Bioreactor for High Purity Mesenchymal Stem Cell Production. PLoS ONE.

[86] Du W., Zhang K., Zhang S., Wang R., Nie Y., Tao H., Han Z., Liang L., Wang D., Liu J. (2017). Enhanced Proangiogenic Potential of Mesenchymal Stem Cell-Derived Exosomes Stimulated by a Nitric Oxide Releasing Polymer. Biomaterials.

[87] Liu L., Liu Y., Feng C., Chang J., Fu R., Wu T., Yu F., Wang X., Xia L., Wu C. (2019). Lithium-Containing Biomaterials Stimulate Bone Marrow Stromal Cell-Derived Exosomal MiR-130a Secretion to Promote Angiogenesis. Biomaterials.

[88] Park D.J., Yun W.S., Kim W.C., Park J.-E., Lee S.H., Ha S., Choi J.S., Key J., Seo Y.J. (2020). Improvement of Stem Cell-Derived Exosome Release Efficiency by Surface-Modified Nanoparticles. J. Nanobiotechnol..

[89] Wu Z., He D., Li H. (2021). Bioglass Enhances the Production of Exosomes and Improves Their Capability of Promoting Vascularization. Bioact. Mater..

[90] Haraszti R.A., Miller R., Dubuke M.L., Rockwell H.E., Coles A.H., Sapp E., Didiot M.-C., Echeverria D., Stoppato M., Sere Y.Y. (2019). Serum Deprivation of Mesenchymal Stem Cells Improves Exosome Activity and Alters Lipid and Protein Composition. iScience.

[91] Gupta S., Rawat S., Arora V., Kottarath S.K., Dinda A.K., Vaishnav P.K., Nayak B., Mohanty S. (2018). An Improvised One-Step Sucrose Cushion Ultracentrifugation Method for Exosome Isolation from Culture Supernatants of Mesenchymal Stem Cells. Stem Cell Res. Ther..

[92] Smith J.T., Wunsch B.H., Dogra N., Ahsen M.E., Lee K., Yadav K.K., Weil R., Pereira M.A., Patel J.V., Duch E.A. (2018). Integrated Nanoscale Deterministic Lateral Displacement Arrays for Separation of Extracellular Vesicles from Clinically-Relevant Volumes of Biological Samples. Lab Chip.

[93] Yu Z., Lin S., Xia F., Liu Y., Zhang D., Wang F., Wang Y., Li Q., Niu J., Cao C. (2021). ExoSD Chips for High-Purity Immunomagnetic Separation and High-Sensitivity Detection of Gastric Cancer Cell-Derived Exosomes. Biosens. Bioelectron..

[94] Heath N., Grant L., De Oliveira T.M., Rowlinson R., Osteikoetxea X., Dekker N., Overman R. (2018). Rapid Isolation and Enrichment of Extracellular Vesicle Preparations Using Anion Exchange Chromatography. Sci. Rep..

[95] Song Z., Mao J., Barrero R.A., Wang P., Zhang F., Wang T. (2020). Development of a CD63 Aptamer for Efficient Cancer Immunochemistry and Immunoaffinity-Based Exosome Isolation. Molecules.

[96] Zhou Z., Chen Y., Qian X. (2022). Target-Specific Exosome Isolation through Aptamer-Based Microfluidics. Biosensors.

[97] Zhang K., Yue Y., Wu S., Liu W., Shi J., Zhang Z. (2019). Rapid Capture and Nondestructive Release of Extracellular Vesicles Using Aptamer-Based Magnetic Isolation. ACS Sens..

[98] Kimiz-Gebologlu I., Oncel S.S. (2022). Exosomes: Large-Scale Production, Isolation, Drug Loading Efficiency, and Biodistribution and Uptake. J. Control. Release.

[99] Gebeyehu A., Kommineni N., Bagde A., Meckes D.G., Sachdeva M.S. (2021). Role of Exosomes for Delivery of Chemotherapeutic Drugs. Crit. Rev. Ther. Drug Carrier Syst..

[100] Zhang Q., Zhang H., Ning T., Liu D., Deng T., Liu R., Bai M., Zhu K., Li J., Fan Q. (2020). Exosome-Delivered c-Met SiRNA Could Reverse Chemoresistance to Cisplatin in Gastric Cancer. Int. J. Nanomed..

[101] Yuan Z., Kolluri K.K., Gowers K.H.C., Janes S.M. (2017). TRAIL Delivery by MSC-Derived Extracellular Vesicles Is an Effective Anticancer Therapy. J. Extracell. Vesicles.

[102] O’Brien K., Lowry M.C., Corcoran C., Martinez V.G., Daly M., Rani S., Gallagher W.M., Radomski M.W., MacLeod R.A.F., O’Driscoll L. (2015). MiR-134 in Extracellular Vesicles Reduces Triple-Negative Breast Cancer Aggression and Increases Drug Sensitivity. Oncotarget.

[103] Yuan Y., Wang Q., Cao F., Han B., Xu L. (2019). MiRNA-134 Suppresses Esophageal Squamous Cell Carcinoma Progression by Targeting FOXM1. Int. J. Clin. Exp. Pathol..

[104] Yang Z., Shi J., Xie J., Wang Y., Sun J., Liu T., Zhao Y., Zhao X., Wang X., Ma Y. (2020). Large-Scale Generation of Functional MRNA-Encapsulating Exosomes via Cellular Nanoporation. Nat. Biomed. Eng..

[105] Nawaz M., Heydarkhan-Hagvall S., Tangruksa B., González-King Garibotti H., Jing Y., Maugeri M., Kohl F., Hultin L., Reyahi A., Camponeschi A. (2023). Lipid Nanoparticles Deliver the Therapeutic VEGFA MRNA In Vitro and In Vivo and Transform Extracellular Vesicles for Their Functional Extensions. Adv. Sci..

[106] Wu M., Ozcelik A., Rufo J., Wang Z., Fang R., Jun Huang T. (2019). Acoustofluidic Separation of Cells and Particles. Microsyst. Nanoeng..

[107] Wang Z., Li F., Rufo J., Chen C., Yang S., Li L., Zhang J., Cheng J., Kim Y., Wu M. (2020). Acoustofluidic Salivary Exosome Isolation. J. Mol. Diagn..

[108] Fan Y., Wang X., Ren J., Lin F., Wu J. (2022). Recent Advances in Acoustofluidic Separation Technology in Biology. Microsyst. Nanoeng..

[109] Lin Y., Wu J., Gu W., Huang Y., Tong Z., Huang L., Tan J. (2018). Exosome–Liposome Hybrid Nanoparticles Deliver CRISPR/Cas9 System in MSCs. Adv. Sci..

[110] Li L., He D., Guo Q., Zhang Z., Ru D., Wang L., Gong K., Liu F., Duan Y., Li H. (2022). Exosome-Liposome Hybrid Nanoparticle Codelivery of TP and MiR497 Conspicuously Overcomes Chemoresistant Ovarian Cancer. J. Nanobiotechnol..

[111] Osteikoetxea X., Silva A., Lázaro-Ibáñez E., Salmond N., Shatnyeva O., Stein J., Schick J., Wren S., Lindgren J., Firth M. (2022). Engineered Cas9 Extracellular Vesicles as a Novel Gene Editing Tool. J. Extracell. Vesicles.

[112] Jafari D., Shajari S., Jafari R., Mardi N., Gomari H., Ganji F., Forouzandeh Moghadam M., Samadikuchaksaraei A. (2020). Designer Exosomes: A New Platform for Biotechnology Therapeutics. BioDrugs.

[113] Sutaria D.S., Jiang J., Elgamal O.A., Pomeroy S.M., Badawi M., Zhu X., Pavlovicz R., Azevedo-Pouly A.C.P., Chalmers J., Li C. (2017). Low Active Loading of Cargo into Engineered Extracellular Vesicles Results in Inefficient MiRNA Mimic Delivery. J. Extracell. Vesicles.

[114] Z L., X Z., M W., X G., L Z., R S., W S., Y D., G Y., L Y. (2019). In Vitro and in Vivo RNA Inhibition by CD9-HuR Functionalized Exosomes Encapsulated with MiRNA or CRISPR/DCas9. Nano Lett..

[115] Antes T.J., Middleton R.C., Luther K.M., Ijichi T., Peck K.A., Liu W.J., Valle J., Echavez A.K., Marbán E. (2018). Targeting Extracellular Vesicles to Injured Tissue Using Membrane Cloaking and Surface Display. J. Nanobiotechnol..

[116] Martins Á.M., Ramos C.C., Freitas D., Reis C.A. (2021). Glycosylation of Cancer Extracellular Vesicles: Capture Strategies, Functional Roles and Potential Clinical Applications. Cells.

[117] Royo F., Cossío U., de Angulo A.R., Llop J., Falcon-Perez J.M. (2019). Modification of the Glycosylation of Extracellular Vesicles Alters Their Biodistribution in Mice. Nanoscale.

[118] Guo J., Wang F., Hu Y., Luo Y., Wei Y., Xu K., Zhang H., Liu H., Bo L., Lv S. (2023). Exosome-Based Bone-Targeting Drug Delivery Alleviates Impaired Osteoblastic Bone Formation and Bone Loss in Inflammatory Bowel Diseases. Cell Rep. Med..

[119] Rana S., Yue S., Stadel D., Zöller M. (2012). Toward Tailored Exosomes: The Exosomal Tetraspanin Web Contributes to Target Cell Selection. Int. J. Biochem. Cell Biol..

[120] Zou J., Shi M., Liu X., Jin C., Xing X., Qiu L., Tan W. (2019). Aptamer-Functionalized Exosomes: Elucidating the Cellular Uptake Mechanism and the Potential for Cancer-Targeted Chemotherapy. Anal. Chem..

[121] Liang G., Zhu Y., Ali D.J., Tian T., Xu H., Si K., Sun B., Chen B., Xiao Z. (2020). Engineered Exosomes for Targeted Co-Delivery of MiR-21 Inhibitor and Chemotherapeutics to Reverse Drug Resistance in Colon Cancer. J. Nanobiotechnol..

[122] Xu X., Liang Y., Li X., Ouyang K., Wang M., Cao T., Li W., Liu J., Xiong J., Li B. (2021). Exosome-Mediated Delivery of Kartogenin for Chondrogenesis of Synovial Fluid-Derived Mesenchymal Stem Cells and Cartilage Regeneration. Biomaterials.

[123] Jia G., Han Y., An Y., Ding Y., He C., Wang X., Tang Q. (2018). NRP-1 Targeted and Cargo-Loaded Exosomes Facilitate Simultaneous Imaging and Therapy of Glioma in vitro and in vivo. Biomaterials.

